# Identification of Autotoxic Compounds in Fibrous Roots of Rehmannia (*Rehmannia glutinosa* Libosch.)

**DOI:** 10.1371/journal.pone.0028806

**Published:** 2012-01-03

**Authors:** Zhen-Fang Li, Yan-Qiu Yang, Dong-Feng Xie, Lan-Fang Zhu, Zi-Guan Zhang, Wen-Xiong Lin

**Affiliations:** Agroecological Institute, Fujian Agriculture and Forestry University, Fuzhou, China; Universidad Nacional Autonoma de Mexico,Instituto de Biotecnologia, Mexico

## Abstract

Rehmannia is a medicinal plant in China. Autotoxicity has been reported to be one of the major problems hindering the consecutive monoculture of Rehmannia. However, potential autotoxins produced by the fibrous roots are less known. In this study, the autotoxicity of these fibrous roots was investigated. Four groups of autotoxic compounds from the aqueous extracts of the fibrous roots were isolated and characterized. The ethyl acetate extracts of these water-soluble compounds were further analyzed and separated into five fractions. Among them, the most autotoxic fraction (Fr 3) was subjected to GC/MS analysis, resulting in 32 identified compounds. Based on literature, nine compounds were selected for testing their autotoxic effects on radicle growth. Seven out of the nine compounds were phenolic, which significantly reduced radicle growth in a concentration-dependent manner. The other two were aliphatic compounds that showed a moderate inhibition effect at three concentrations. Concentration of these compounds in soil samples was determined by HPLC. Furthermore, the autotoxic compounds were also found in the top soil of the commercially cultivated Rehmannia fields. It appears that a close link exists between the autotoxic effects on the seedlings and the compounds extracted from fibrous roots of Rehmannia.

## Introduction

Rehmannia (*Rehmannia glutinosa* Libosch) is in the Scrophulariaceae family and is one of the most common and important medicinal herbal plants in China. It is perennial and its fresh or dried tuberous roots are used as a high demand traditional Chinese medicinal ingredient for hematologic conditions, sedation, insomnia and diabetes [1], [2]. Its commercial cultivation has been practiced for almost 1500 years in China. However, the consecutively monocultured plants are prone to severe diseases resulting in reduced biomass, especially the tuberous products. To maintain the cultivation, the farmers commonly limited the cultivation on a same plot once every eight years. Therefore, less desirable areas outside Jiaozuo had to be used for the planting with decreased tuber yields and lower product quality [3].

The autotoxicity issue has attracted much attention [4], [5]. Autotoxicity is the phenomenon whereby mature plants inhibit the growth of their own seedlings through the release of autotoxic chemicals. It has been found to exist in various crops [6], [7], such as greenhouse crops [8], [9], fruits [10], [11], forage [12], [13], horticultural and medicinal plants [4], [5], [14], [15], [16]. Several groups of chemicals have been implicated in autotoxicity, including terpenoids, phenolics, steroids, alkaloids, and cyanogenic glycosides. Recently, autotoxicity in Rehmannia has been reported [15],[16],[17] especially in relation to the compounds derived from the root exudates. However, to date, the degradation of fibrous roots and its products had not been studied, and the mechanism of autotoxicity in Rehmannia remains unknown.

This study aims to identify substances that contribute directly to Rehmannia autotoxicity. A number of potentially autotoxic compounds from the fibrous roots were isolated and characterized. The inhibitory effect of these compounds on seedling growth was observed. Furthermore, the concentration of these bioactive compounds in the top soil collected from one-year cultivated and two-year consecutively moncultured Rehmannia fields was determined.

## Materials and Methods

### Sample collection and autotoxic compound extraction

#### Water extraction

The fields were located in Jiao-zuo County (113°21′E, 35°24′N), He-nan province of China, which is the optimal production areas of Rehmannia. The samples were collected in October 2008 (Figure S1).

Fibrous roots of one-year cultivated Rehmannia plants at the mature stage were collected. The air-dried roots (500 g), passed 2 mm sieve, were soaked in 1000 mL distilled water at 25–30°C for 48 h. The extract was filtered, and the extraction was repeated three times. The aqueous extracts from the three extractions were combined and concentrated to 20 mL under vacuum at 50°C, then freeze-dried under liquid N_2_ at −180°C Approximately 530 mg of the dried material were obtained from the 500 g of air-dried fibrous roots.

Top soil samples (20 cm depth) were collected from both one-year cultivated and two-year consecutively moncultured Rehmannia fields in Jiaozuo county at harvest time. A soil sample from an adjacent uncultivated field was collected as a control. Potential autotoxic compounds were extracted from the soil samples using the same method for the fibrous roots. Approximately 400 mg of dried material were obtained from the 500 g air-dried soil samples.

#### Ethanol extraction and partitioning

Air-dried fibrous roots (2 kg) were extracted with 95% ethanol (5 L) at room temperature for 5 d. This process was repeated once. The extract was concentrated by evaporation to 200 mL at 50°C under vacuum followed by freeze-drying under liquid N_2_ (−180°C), and then dissolved in 200 mL distilled water.

**Figure 1:**
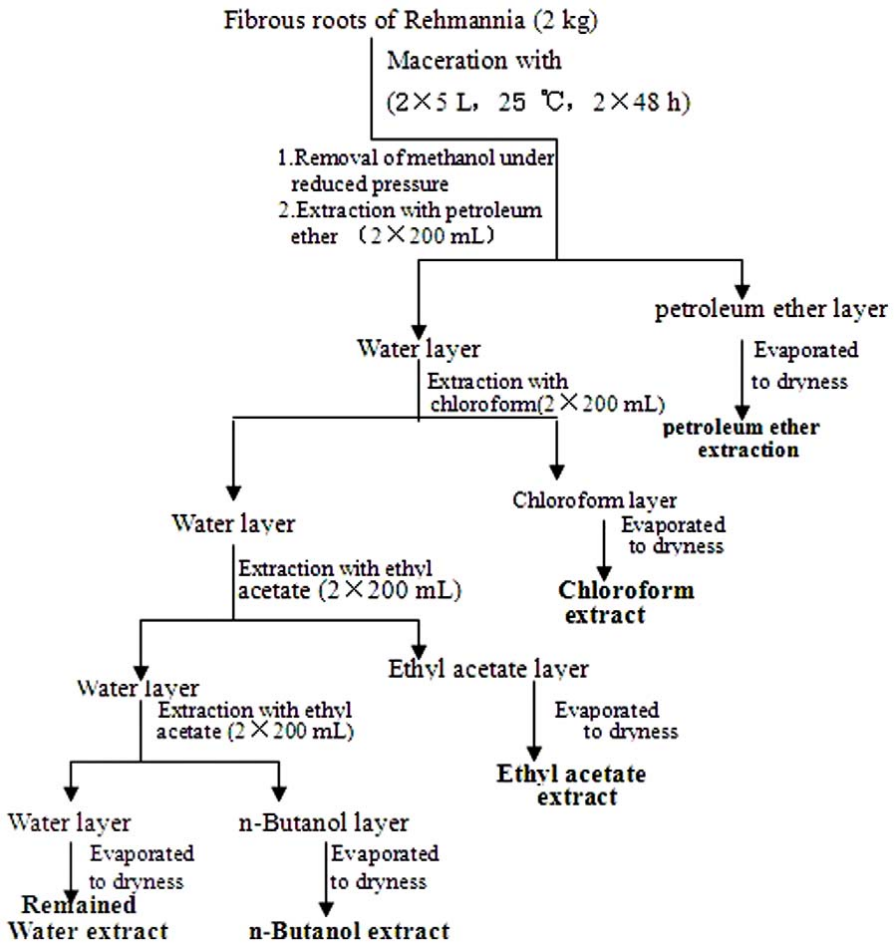
Flow chart of extraction procedures.

The aqueous solution was consecutively partitioned with petroleum ether, chloroform, ethyl acetate, and n-butanol, as shown in Figure 1. The solvent extractions were performed by shaking in separation funnels for 10 min, followed by evaporation under vacuum.

### Bioassays and statistical analysis

To examine the functional effect of the potential autotoxic compounds on Rehmannia growth, measurements of Rehmannia radicles in petri dishes was performed. The extracts of petroleum ether, chloroform, ethyl acetate, n-butanol and the ethyl acetate-extracted fractions (1–5), of fibrous roots, monocultured soils for 1 year and 2 years, and uncultivated soil, were diluted with distilled water into 2, 5, 10, 20 and 50 mg L^−1^. 5 mL of each of the diluted solutions were transferred into the 10-cm diameter petri dishes containing double-layered filter paper (Whatman No. 42). In addition, individual compounds, including 7 aliphatic and 2 phenolics as identified in Fr3, were dissolved in a small volume of methanol and transferred onto the double-layered filter paper in the petri dishes. The solvents were evaporated in a draft chamber for 1 h. The filter paper containing these compounds was moistened with 5 mL distilled water. The final concentrations of the compounds in water were 2, 10, and 50 mg L^−1^, while distilled water was used as control. Twenty Rehmannia seeds were placed on the filter paper in the petri dishes. All dishes were maintained in a tissue culture room at 26°C with fluorescent lights for 11 h (8∶00–20∶00) as described previously [7]. The fluorescent light intensity was 4.17±0.18×103 lux. Germinated seeds with >1 mm radius were recorded and the radicle lengths were measured after incubation for 5 d.

The radicle lengths of the treated ones in comparison with the controls were used as an index of the inhibition rates (IRs). IR was calculated as: IR  =  (control - treatment)/control×100%. When IR is greater than 0, it indicates an inhibition; conversely when IR is less than 0, it indicates promoting growth [18]. All data were subject to an analysis of variance using the Statistical Analysis System Program (SPSS). Each value was expressed as the mean of three replicates±standard error (SE).

### Identification of autotoxic chemicals

The ethyl acetate extract was further analyzed. About 8 g of the extract was subjected to column chromatography (CC), packed with silica gel (200–300 mesh) and eluted with chloroform (CHCl_3_)-methanol (MeOH), followed by a gradient solvent system (0–100 MeOH) to yield five fractions (Fr1-5). The fractions were analyzed by thin-layer chromatography (TLC) on the same Rf value. Preliminary bioassay study indicated that fraction 3 (Fr3) exhibited the greatest inhibitory activity on radicle growth of Rehmannia. Thus, Fr3 was further purified using the reversed phase multi-purpose GC column chromatography (Varian, California, U.S.A., VF-5 ms, 30 m×0.25 mm×0.25 µm).

The GC/MS fingerprints of Fr3 were obtained with a GC/MS (Autospec-240 MS Ion Trap mass spectroscopy, Varian, California, U.S.A.). Fraction 3 was subsequently dissolved into 2 mL redistilled MeOH. One µL aliquot of this solution was evaporated under a stream of helium to remove residual water. A mixture of 10 µL redistilled MeOH and 10 µL N, O-Bis (trimethylsilyl) trifluoro acetamide (BSTFA) + 1% trimethylchlorosilane (TMCS) was added to the residue to produce trimethylsilyl derivatives by heating at 100°C for 1 h. The solution was filtered through a 0.45 µm filter before injected into the GC-MS system.

The injection volume was 1 µL. Helium was used as the carrier gas, and the flow rate was adjusted to 10 mL min^−1^. The oven temperature was initially programmed at 50°C and ramped to 270°C at a rate of 5°C·min^−1^, where it remained for 10 min. MS data were acquired in the negative ionization mode. The full scan mass covered a range from m/z 50 to 1500. The m/z values for standard autotoxins and the compounds as available in the literature were used to match with those obtained in the spectra for the study.

### HPLC analysis

To determine whether the autotoxic compounds identified in Rehmannia root extracts also existed in ground soil, we tested the soil samples.

The concentration of autotoxic compounds in soil samples was determined using a Waters HPLC system (Alliance HPLC system Massachusetts, U.S.A.).The chromatographic system consisting of a Waters 2695 HPLC system with a reversed-phase column Zorbax Eclipse XDB –C18 (250 mm×4.6 mm, 5 µm column) was used at a flow rate of 1.0 mL min^−1^. The solvent system was a linear gradient of solvent A (methanol) and solvent B (0.5 mol L^−1^ acetic acid in water): from 1% to 25% A in 0–10.0 min, and hold at 25% A for 5 min;from 25% to 80% A in 15.0–25.0 min, and hold at 80% A for 5 min;from 80% to 25% A in 30.0–40.0 min, and hold at 25% A for 10 min. The injected volume was 10 mL of a water solution of the extracts (10 mg mL^−1^). The column temperature was maintained at 35°C. The UV detector was performed at 280 nm. The chromatographic data were recorded and processed with a Waters empower workstation.

HPLC grade acetonitrile, acetic acid (Merck, Darmstadt, Germany), and filtered bi-distilled water, were used for HPLC analysis. The methanol used for extraction was from “Honeywell International” (New Jersey, U.S.A.). Standards of phenolic acids, including (gallic acid, 4-hydroxybenzoic acid, vanillic acid, protocatechuic acid, ferulic acid, benzoic acid, and salicylic acid,) were purchased from Sigma Chemicals Co., U.S.A.. Solvents and standards of phenolic acids were chromatographic grade. The specific recovery rates (%) for the standards were: gallic acid, 96.24±4.42;4-Hydroxybenzoic acid, 92.26±5.35; vanillic acid, 92.15±3.22; protocatechuic acid, 92.44±5.06; ferulic acid, 93.25±5.31; benzoic acid, 90.01±4.38; and salicylic acid, 90.06±3.21.

The dry materials from the water extract were dissolved into 100 mL methanol and passed through a 0.22 µm glass fiber sieve. 7 Phenolic acids compounds found in the samples extracts were dentified by matching the retention time and their spectral characteristics against those of standards. The separation procedures were repeated six times for each standard compound, and data were presented as mean±SE.

## Results

### Bioassay of fibrous root extracts

When compared with the root exudates and the extracts of three different soils (i.e. control soil, one-year monocultured and two-year monocultured soils), the autotoxic compounds extracted from the fibrous roots showed greater inhibitive effects on the radicle growth of Rehmannia (Table 1). The inhibition rate increased with higher concentrations of the extracts. The IR peaked at 50 mg·L^−1^ with 75.76% reduction in seedling radicles (P<0.01; Table 1). A similar trend was found in the soil extracts, but their inhibitory effects were always lower than those of the fibrous roots'. The result indicated that the chemical compounds extracted from the fibrous roots had a major auto-inhibitory effect on the growth of Rehmannia radicle.

**Table 1:** Inhibition effect of aqueous extracts from soil and fibrous roots on Rehmannia radicles growth.

	Uncultivated (control soil)		Monocultured soil (1-year)		Monocultured soil (2-year)		Fibrous roots	
Concentration	Radicle length (mm)	IR (%)	Radicle length (mm)	IR (%)	Radicle length (mm)	IR (%)	Radicle length (mm)	IR (%)
**2 mg·L^−1^**	9.67±0.23a	-	9.44±0.09b	−3.09	9.22±0.18c	−5.15	8.68±0.14d	−10.31
**5 mg·L^−1^**	10.12±0.07a	-	8.37±0.21b	−16.83	8.04±0.16c	−20.79	7.46±0.25d	−25.74
**10 mg·L^−1^**	9.33±0.27a	-	6.67±0.08b	−27.96	5.14±0.16c	−45.16	4.92±0.28c	−47.31
**20 mg·L^−1^**	9.07±0.23a	-	5.45±0.24b	−39.56	4.31±0.13c	−52.75	3.23±0.15c	−64.84
**50 mg·L^−1^**	9.92±0.30a	-	2.86±0.14b	−70.71	2.53±0.21b	−74.75	2.35±0.24b	−75.76

Note: Values on the same row followed by the same lowercase letters are not statistically different at P = 0.05 by Duncan's test, the same below.

### Bioassay of partitioned compounds of fibrous root extracts

The bioassay with petroleum ether, chloroform, ethyl acetate, and n-butanol partitions showed that the four extracts exhibited dose-dependent inhibition effects on the radicle growth and that the ethyl acetate extract had the greatest inhibitory effect (Table 2). At the concentration of 50 mg L^−1^, the inhibition rates of petroleum ether, chloroform, ethyl acetate, and n-butanol extracts were 8.7%, 5.8%, 64.2%, and 25.8%, respectively.

**Table 2:** Inhibitory effect on the growth of Rehmannia radicles when exposed to fibrous root extracts.

	Radicle length of Rehmannia (mm)				
Fraction	2 mg·L^−1^	5 mg·L^−1^	10 mg·L^−1^	20 mg·L^−1^	50 mg·L^−1^
**Distilled water (control)**	9.89±0.16b	9.89±0.16b	9.89±0.16a	9.89±0.16a	9.89±0.16a
**Petroleum ether extract**	10.06±0.14a	10.14±0.21a	9.19±0.18b	9.06±0.24c	9.03±0.12b
**Chloroform extract**	10.13±0.12a	10.14±0.16a	10.12±0.13a	9.68±0.12b	9.32±0.16b
Ethyl **acetate extract**	8.67±0.09d	7.85±0.24d	4.79±0.23d	4.32±0.16e	3.54±0.14d
**n-Butanol extract**	9.35±0.25c	9.11±0.15c	8.17±0.16c	8.31±0.23d	7.34±0.17c

### Bioassay of column chromatography fractions of ethyl acetate extract

Bioassays with each of the five fractions from ethyl acetate extract showed a significant reduction in the length of the radicle in the presence of Fr3 (P<0.01; Table 3). When the concentration was greater than 20 mg L^−1^, the reduction reached 100%. This indicates that the chemical compounds in Fr3 had a major auto-inhibitory effect on the seedling growth, and so Fr3 was considered a candidate for further identification and characterization.

**Table 3:** Effect of Fractions 1–5 from Rehmannia fibrous root's ethyl acetate extract on radicle growth of Rehmannia.

	Radicle Length of Rehmannia (mm)				
Fraction	2 mg·L^−1^	5 mg·L^−1^	10 mg·L^−1^	20 mg·L^−1^	50 mg·L^−1^
Fr 1	9.37±0.12a	10.11±0.25a	9.34±0.09a	9.22±0.13a	9.13±0.17b
Fr 2	7.06±0.09c	6.10±0.14d	6.53±0.12c	6.06±0.15c	6.03±0.15d
Fr 3	4.37±0.23e	3.15±0.27e	2.79±0.18d	0 d	0 f
Fr 4	6.15±0.16d	6.62±0.18c	6.13±0.14c	5.65±0.18c	5.33±0.14e
Fr 5	8.39±0.13b	8.31±0.15b	8.17±0.21b	8.08±0.20b	7.96±0.22c
Sterilized water (control)	9.67±0.23a	9.67±0.23a	9.67±0.23a	9.67±0.23a	9.67±0.23a

### Autotoxic effect of compounds identified in fraction 3

**Figure 2:**
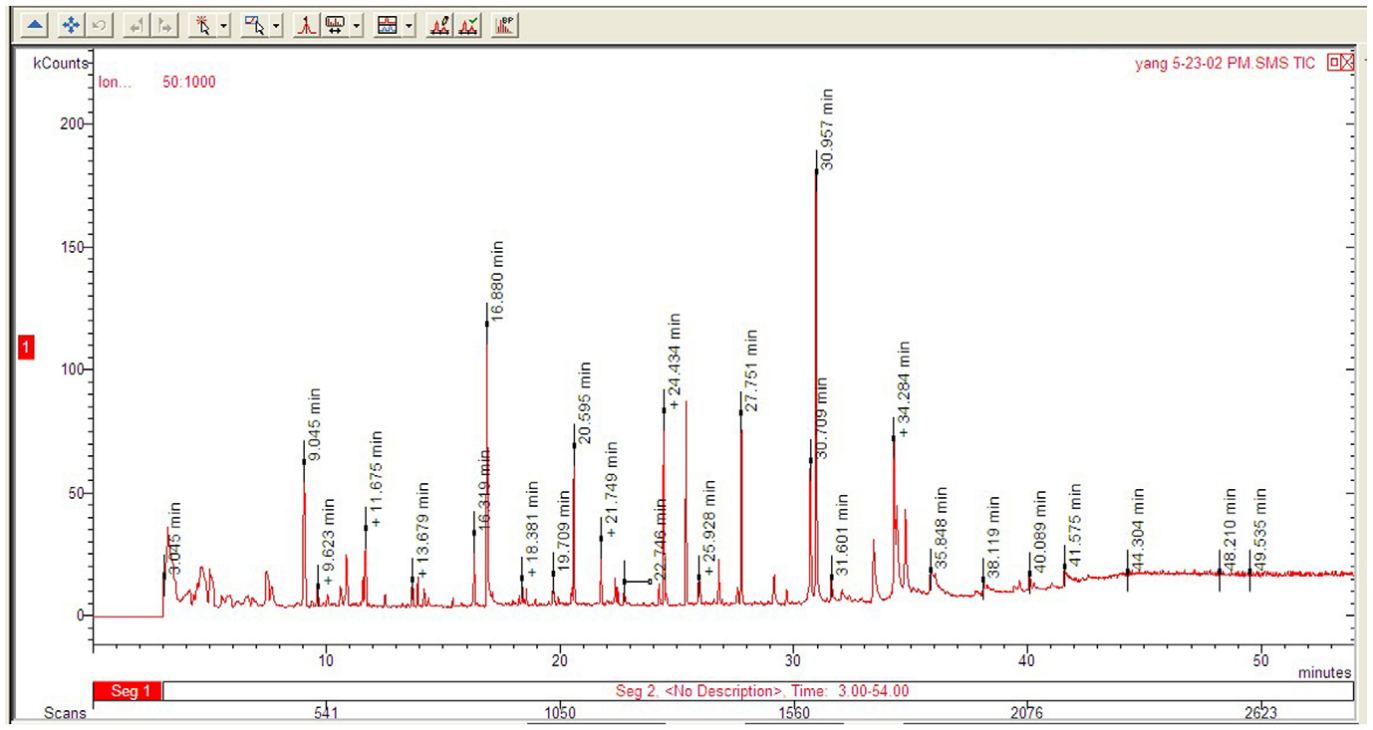
Total ion chromatogram of bioactive Fraction 3 of ethyl acetate extract from Rehmannia fibrous roots.

By comparing with a GC/MS user-library spectrum of pure reference compounds, GC/MS analysis for Fr3 identified a total of 32 compounds in Fr3. They were classified into 5 groups: phenolics, aliphatic compounds, terpenoids, steroids, and others (Figure 2, Table 4).

**Table 4:** Compounds in bioactive Fraction 3 of ethyl acetate extract from Rehmannia fibrous roots as identified by GC/MS analysis.

Rt	CAS	Scientific name	Formula
**Aliphatic compounds**			
22.746	5870-93-9	Butanoic acid, heptyl ester	C_11_H_22_O_2_
20.595	544-63-8	Tetradecanoic acid	C_14_H_28_O_2_
18.381	7132-64-1	Pentadecanoic acid, methyl ester	C_16_H_32_O_2_
16.319	109-52-4	Pentanoic acid	C_5_H_10_O_2_
31.601	143-07-7	Dodecanoic acid	C_12_H_24_O_2_
33.410	55000-42-5	11-Hexadecenoic acid, methyl ester	C_17_H_32_O_2_
40.089	112-95-8	Eicosane	C_20_H_42_
35.848	544-35-4	Linoleic acid ethyl ester	C_20_H_36_O_2_
41.575	112-80-1	Oleic Acid	C_18_H_34_O_2_
**Phenolic compounds**			
30.709	496-16-2	Benzofuran, 2,3-dihydro-	C_8_H_8_O
30.957	1135-24-6	Ferulic acid	C_10_H_10_O_4_
19.709	6781-42-6	1,1′-(1,3-Phenylene)diethanone	C_10_H_10_O_2_
25.928	876-02-8	4-Hydroxy-3-methylacetophenone	C_9_H_10_O_2_
25.673	121-33-5	Vanillin	C_8_H_8_O_3_
24.434	69-72-7	Salicylic acid	C_7_H_6_O_3_
27.751	149-91-7	Gallic acid	C_7_H_6_O_5_
34.284	99-50-3	Protocatechuic acid	C_7_H_6_O_4_
35.132	99-96-7	4-Hydroxybenzoic acid	C_7_H_6_O_3_
21.673	65-85-0	Benzoic acid	C_7_H_6_O_2_
**Terpene**			
11.023	933-40-4	Cyclohexane, 1,1-dimethoxy-	C_8_H_16_O_2_
11.675	109119-91-7	Aromadendrene	C_15_H_24_
13.679	135760-25-7	Ascaridole epoxide	C_10_H_16_O_3_
15.567	77-53-2	Cedrol	C_15_H_26_O
Steroids			
14.098	546-97-4	Columbin	C_20_H_22_O_6_
24.312	61834-65-9	Allopregnane-3,7,11,20-tetra-one	C_21_H_28_O_4_
26.113	17673-25-5	Phorbol	C_20_H_28_O_6_
27.067	52-21-1	Prednisolone Acetate	C_23_H_30_O_6_
**Others**			
9.045	60485-45-2	Santolina epoxide	C_10_H_16_O
12.554	110-15-6	Butanedioic acid	C_4_H_6_O_4_
16.880	97-67-6	L-Hydroxybutanedioic acid	C_4_H_6_O_5_
18.607	86-73-7	Fluorene	C_13_H_10_
22.547	84-66-2	Phthalic acid	C_12_H_14_O_4_

Previous studies suggested that several chemicals detected in the bioactive Fr3 were possibly allelopathic compounds, which were reported as allelochemicals in other crops [4],[8],[19]. Based on previous reports and the availability of the chemicals in Fr3, nine individual compounds were selected to test their potential autotoxic effects on Rehmannia radicle growth (Table 5). Among the 9 chemicals examined, seven phenolic compounds showed dose-respondent inhibitory effects on the seedling growth. Specifically, the radicle of the seedlings reduced significantly by 45–76% when they were treated with the phenolic compounds (except salicylic acid) at a concentration of 50 mg·L^−1^ (P<0.01). Ferulic acid and vanillin acid were the most potent compounds in the test, and therefore, they were considered as the potential allelochemicals. All of the compounds inhibited the seedling growth even at a low concentration (*e.g.*, 2 mg·L^−1^, P<0.01), and IRs increased with increasing concentrations. Similarly, the compounds in the aliphatic acid family (e.g., tetradecanoic acid and oleic acid) exhibited dose-respondent inhibitory effects on the seedling growth. The extent of inhibition was around 25% for each compound (Table 5). The results demonstrate clearly that the compounds extracted from the roots of Rehmannia are potent inhibitors on seedling growth. In addition, both the phenolic acid and aliphatic acid compounds might contribute to the autotoxicity of Rehmannia.

**Table 5:** Effect of autotoxic chemicals on radicle growth of Rehmannia.

	Treated with 2 mg·L^−1^		Treated with 2 mg·L^−1^		Treated with 2 mg·L^−1^	
Autotoxic chemicals	Radicle Length (mm)	IR (%)	Radicle Length (mm)	IR (%)	Radicle Length (mm)	IR (%)
Ferulic acid	5.15±0.12e	−46.74	3.79±0.16e	−60.89	2.37±0.17e	−75.50
**Vanillic acid**	5.32±0.14e	−44.98	4.33±0.23e	−55.31	2.62±0.09e	−72.91
**4-Hydroxybenzoic acid**	5.25±0.12e	−45.71	3.84±0.09e	−60.37	2.67±0.16e	−72.39
**Protocatechuic acid**	6.56±0.17d	−32.16	4.92±0.23e	−49.22	3.87±0.13d	−59.98
**Benzoic acid**	6.34±0.21d	−34.44	5.26±0.11d	−45.72	4.13±0.17d	−57.29
**Gallic acid**	7.56±0.11c	−21.82	7.11±0.16c	−26.63	5.32±0.20c	−44.98
**Salicylic acid**	8.38±0.15b	13.34	8.13±0.13b	−16.10	7.33±0.14b	−24.36
**Oleic acid**	7.65±0.14c	−20.89	7.43±0.12c	−23.32	6.89±0.18b	−28.75
Tetradecanoic acid	8.53±0.22b	−11.79	7.42±0.09c	−23.43	7.01±0.20b	−27.51
**Sterilized water (control)**	9.69±0.22a	-	9.69±0.22a	-	9.69±0.22a	-

### Identification of autotoxic compounds in soil samples

**Figure 3:**
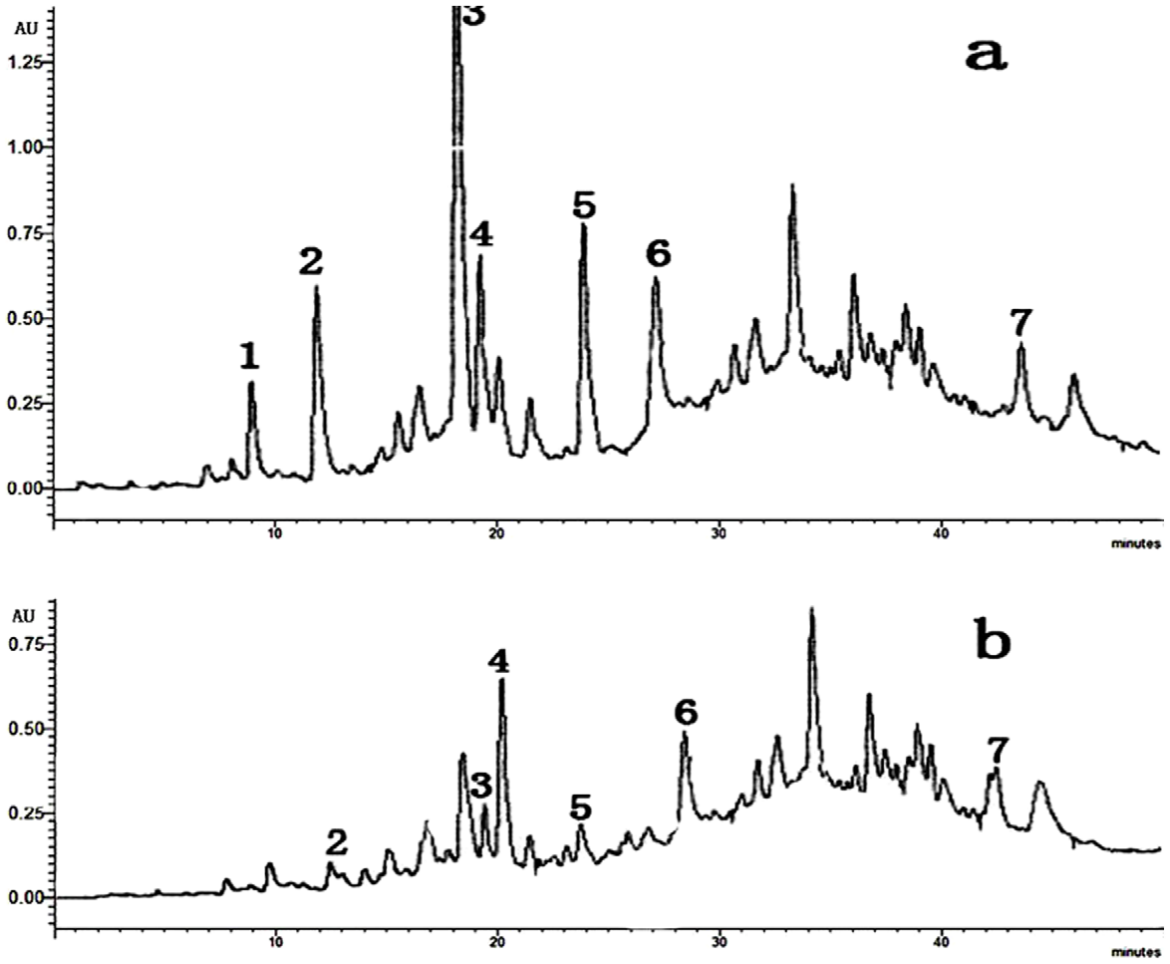
HPLC chromatograms of two-year consecutively monocultured soil (a), one-year cultivated soil (b). Compounds detected from samples in order of appearance in eluant: gallic acid (1), protocatechuic acid (2), 4-hydroxybenzoic acid (3), vanillic acid (4), salicylic acid (5), ferulic acid (6), and benzoic acid (7).

In the fields where Rehmannia were monocultivated for 1 year and 2 years, seven phenolic compounds identified as the potential autotoxins were found at different concentrations (Table 6, Figure 3), 4-hydroxybenzoic acid being the most abundant. In the control and 1-year monocultured soil samples, only 6 phenolic compounds were detected. Thus, the findings presented here indicate that the compounds identified in Rehmannia roots can be found in relative abundance in soils previously cultivated with Rehmannia, whereas they are absent or present at much lower concentrations in non-cultivated soils. Furthermore, the higher inhibitory chemical concentration in the consecutively monocultured soil than in the newly planted soil was due to the accumulation of autotoxic compounds in the rhizosphere soil.

**Table 6:** Concentration of autotoxic compounds soil samples.

	Control Soil		one-year cultivated soil		two-year moncultured soil	
Autotoxic chemicals	Rt (min)	Conc. (mg·kg^−1^)	Rt (min)	Conc. (mg·kg^−1^)	Rt (min)	Conc. (mg·kg^−1^)
**Gallic acid**	-	-	-	-	9.316	2.586
**Protocatechuic acid**	12.132	0.171	12.325	0.804	12.239	4.825
**4-Hydroxybenzoic acid**	18.412	3.653	18.447	11.757	18.236	12.209
**Vanillic acid**	22.211	0.622	20.534	1.019	20.426	5.279
**Salicylic acid**	24.011	0.628	24.194	7.036	23.897	8.829
Ferulic acid	28.226	2.946	28.412	3.624	28.131	8.641
**Benzoic acid**	42.865	1.880	42.813	3.026	44.001	3.315

## Discussion

Our results demonstrate that the compounds isolated from ethyl acetate-soluble extracts of Rehmannia fibrous roots had the most auto-inhibitory effects on the seedling growth. Specifically, 32 chemicals were identified by GC/MS that included 9 aliphatic, 10 phenolic, 4 terpene, 4 steroids and 5 other compounds. Among them, the 7 phenolic compounds and 2 aliphatic acids selected for testing the inhibition effects showed a significant suppressive function on the seedling growth. The inhibitory effects were somewhat related to the concentration of the autotoxins. More importantly, all 7 bioactive phenolic compounds were detected in the top soil of the Rehmannia fields. It appeared that our study provided the first direct evidence that the autotoxic chemicals detected in the soils of different-year consecutively monocultured Rehmannia fields could be traced back to the roots of Rehmannia. During soil sample collection, we noticed that a large amount of fibrous root waste was left in the soil after harvest. It is likely that the autotoxic compounds found in soils were derived partly from the root exudates or the degraded plant tissues. Once released into the soil and allowed to accumulate, these compounds might play a major role in the autotoxic effects on the seedling growth.

In this study, we found that the inhibitory effect of each single compound was not as potent as the bioactive Fr3 (which contained all compounds). It might result from the additive or synergistic effect of the mixture compounds extracted from Rehmannia fibrous roots and its rhizosphere soil. The similar results were reported in the case of other plants [20],[21].

The consecutive monoculture problems in the case study were also defined as “replanting disease” or “sick soil syndrome”, and it is a very common phenomenon in many fruit trees, such as apples, pears, and plums. A wide variety of tree pathogens, including bacteria, fungi, nematodes, and viruses, have been linked to the “replanting disease” in fruit trees. These pathogens may not be harmful to the mature trees, yet they retard the growth of young trees in the same field [22]. It has been reported that the presence of fungal pathogens in soils contributes to the “replanting disease” of Rehmannia [23],[24]. However, this study provides evidence that the autotoxicity is another major cause of the disease [4]. Identification of the autotoxic compounds in this study might be helpful to further understand the problems associated with consecutive monoculture of Rehmannia, and it was also conducive to make the solution to effectively control the “replanting disease” for Rehmannia in consecutively cropping sequence.

## Supporting Information

Figure S1 **The field study picture.**
(DOC)
