# Supplementary figures and images for: Identification of Autotoxic Compounds in Fibrous Roots of Rehmannia (Rehmannia glutinosa Libosch.)
Source: PLoS One. 2012 Jan 3;7(1):e28806. doi: 10.1371/journal.pone.0028806 (PMC3250401; doi:10.1371/journal.pone.0028806)

**Figure S1. The field study picture**

**
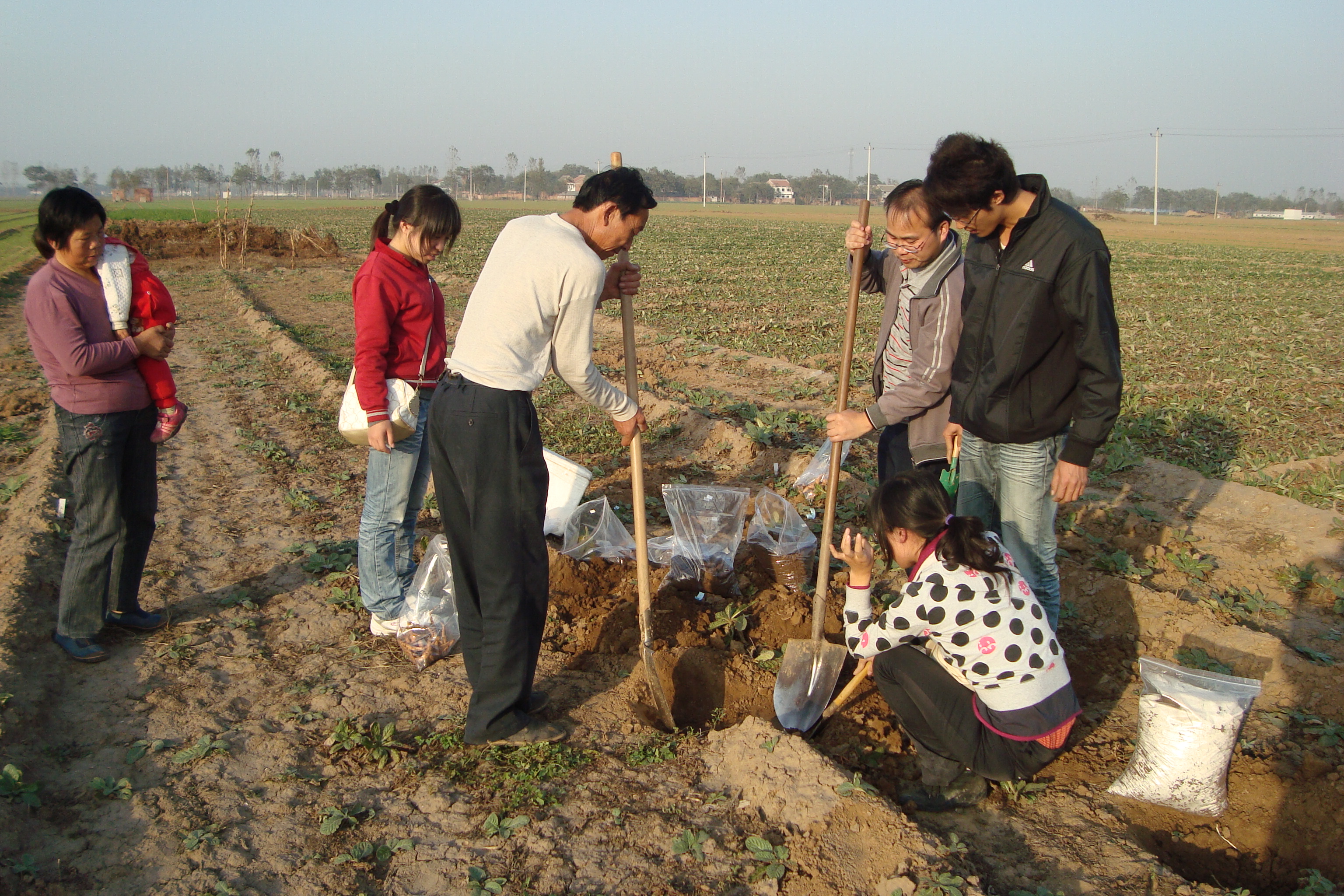
**

Supplement: Figure S1 — The field study picture. (DOC) [file pone.0028806.s001.doc]
